# Breast findings incidentally detected on body MRI

**DOI:** 10.1186/s40064-016-2343-x

**Published:** 2016-06-18

**Authors:** Bianca Bignotti, Giulia Succio, Francesca Nosenzo, Michela Perinetti, Licia Gristina, Stella Barbagallo, Lucia Secondini, Massimo Calabrese, Alberto Tagliafico

**Affiliations:** Department of Health Sciences (DISSAL), University of Genova, Via A. Pastore 1, 16132 Genoa, Italy; Department of Diagnostic Senology, IRCCS Azienda Ospedaliera Universitaria San Martino IST-Istituto Nazionale per la Ricerca sul Cancro, Largo Rosanna Benzi 10, 16132 Genoa, Italy; Institute of Anatomy, Department of Experimental Medicine, University of Genova, Via Leon Battista Alberti, 2, 16132 Genoa, Italy

**Keywords:** Breast, Magnetic resonance imaging, Body, Cancer, Costs

## Abstract

**Objectives:**

To evaluate breast findings incidentally detected on body MRI.

**Methods:**

A retrospective review of the institutional database identified 1752 body MRI performed between January 2015 and September 2015. MRI of women with breast tissue visible in the field-of-view were reviewed for breast findings. Breast findings were classified with the breast imaging reporting and data system (BI-RADS) lexicon. The standard statistic, costs of additional work-up, and the clinical relevance were used to describe breast findings, and we calculated 95 % exact confidence intervals (CIs).

**Results:**

440 body MRI of 440 women (mean age: 57 ± 20 years) included breast tissue in the field-of-view. A total of 41 breast findings were identified in 41 patients. Breast findings were classified BI-RADS 2 N = 25, BI-RADS 3 N = 13, BI-RADS 4 N = 3. A total of 3.6 % [95 % CI 1.6 %, 5.6 %] women with breast tissue visible on MRI had a recommendation for further imaging work-up for a breast finding. The 18.7 % (3 of 16) of these patients had a clinically important finding (breast cancer). Further imaging evaluation increased costs of €108.3 per patient with a breast finding.

**Conclusions:**

Clinically important breast findings could be detected on body MRI in up to 0.7 % (3 of 440) of women.

## Background

Body magnetic resonance imaging (MRI) is a highly sensitive imaging method to assess morphological structures and the presence of a lesion (Schmidt et al. 2013). Since there has been an increased use of MRI in clinical practice, an increased number of incidental findings are expected (Chernyak et al. 2015; Wagner and Aron 2012; Sebastian et al. 2013; Heller et al. 2013; Khosa et al. 2013; Patel et al. 2013). Indeed, incidental findings are increasingly common, and the American College of Radiology developed guidelines for management of incidental findings detected during abdominal and pelvic MRI or computed tomography (CT) (Sebastian et al. 2013; Heller et al. 2013; Khosa et al. 2013; Patel et al. 2013). In addition, several studies evaluated incidental findings outside the region-of-interest during MRI of the heart, breast and spine, using an expanded field-of-view (FOV) (Wyttenbach et al. 2012; Bazzocchi et al. 2012; Dewey et al. 2007; Maxwell et al. 2015). Regarding MRI of the abdomen and the chest (body MRI), different field-of-views are used, based on the clinical suspicious and the specific region-of-interest to be evaluated. In the most frequent clinical indications of body MRI, the anteroposterior size of FOV is approximately 30 centimetres (Erturk et al. 2009; Chang et al. 2008; Lee et al. 2014). Therefore, some breast tissue could be included in body MR examinations, and consequently, some incidental breast findings could be potentially detected. In literature, incidental breast findings have been evaluated and described on CT and (18) F-fluorodeoxyglucose (FDG) positron emission tomography (PET)–CT (Bach et al. 2013; Monzawa et al. 2013; Benveniste et al. 2015). In a population of patients with known non-mammary malignancies, 6 % of incidental breast lesions detected on FDG PET/CT were primary breast cancer (Benveniste et al. 2015). Several studies evaluated potentially relevant incidental findings on MRI, including breast findings, but all of these studies dealt with whole-body MRI and not with more tailored MR examinations (Cieszanowski et al. 2014; Tarnoki et al. 2015). To the best of our knowledge, there are no studies in literature dealing with breast findings detected on body (abdominal and chest) MRI. Therefore, the aim of our study was to evaluate breast findings incidentally detected on body MRI, recommendations for further imaging work-up, and the clinical relevance and health care costs.

## Methods

**Fig. 1:**
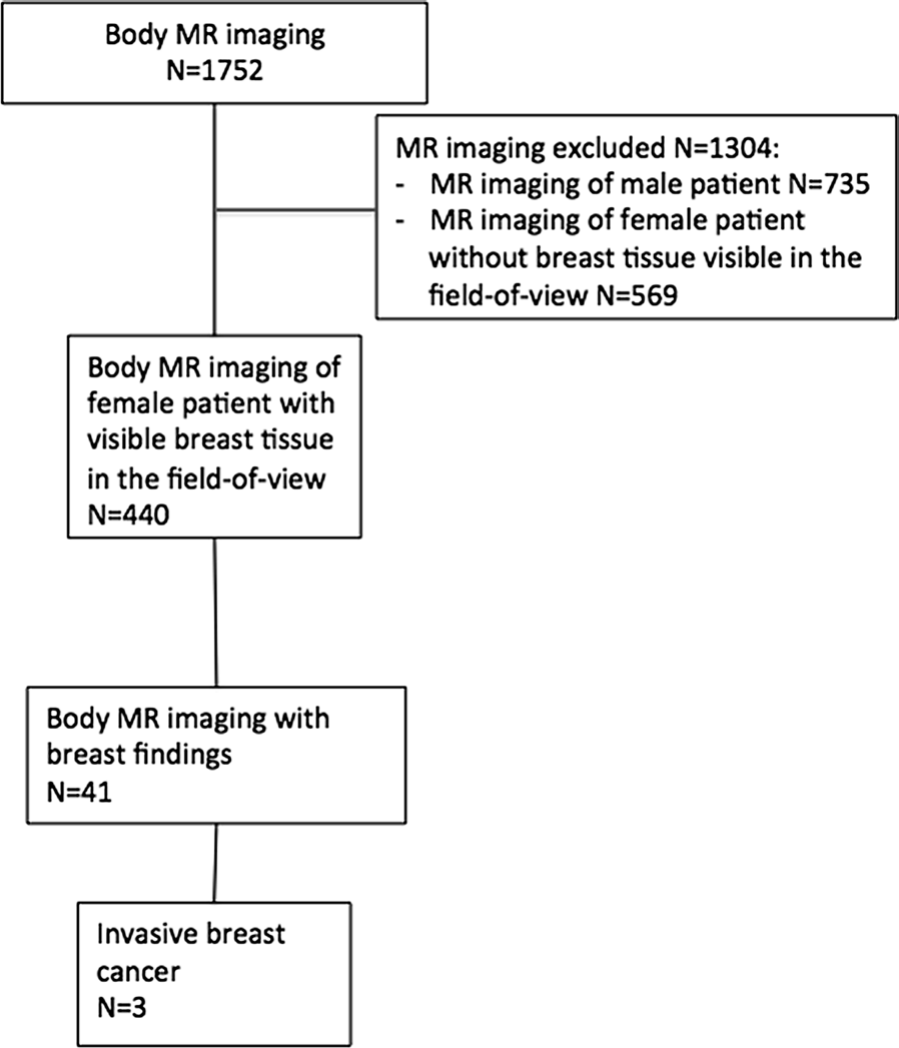
Flow-chart of the study design

This study was notified to the Institutional Review Board, and the requirement for informed consent was waived. Our Centre is a Tertiary Centre with more than 6.000 MRI examinations performed in a year and more than 2.000 are body MRI examinations. Figure 1 shows the flow-chart of the study.

A retrospective query of our body MRI database was performed by seven radiologists in March 2016. The seven radiologists (B.B., G.S., F.N., M.P., L.G., S.B., L.S.) had more than 3 years of experience in body and breast imaging. The retrospective query included the review of the images of body MRI with breast tissue visible on abdominal and chest MRI. The query was done between January 2015 and September 2015. At least 6-months follow-up period of the lesions classified as benign was warranted. For patients who underwent more than one body MRI during the time interval, we recorded each MRI as a separate event. Dedicated breast, musculoskeletal and MR neuro imaging examinations were excluded from the investigation.

A total of 1752 body MRI examinations were reviewed. MRI examinations of male patients and with no breast tissue visible were excluded. Breast findings were evaluated on the remaining body MRI in female patients with breast tissue visible.

For each body MRI examination with breast tissue visible, the percentage of breast tissue visible was recorded and visually estimated as more or less than 50 % of the total breast volume. In addition, patient age, magnetic field where the MRI was performed and MRI type (abdominal or chest) were recorded. In particular, the FOV size and the type of the contrast agent used and the presence or absence of diffusion-weighted images were recorded.

### MRI technique and image interpretation

Abdominal and chest MRI were performed with the patient in a supine position by using a 1.5 T (Magnetom Avanto, Siemens, Germany) or a 3.0 T (GE Signa HDx 3.0T, General Electric Medical Systems, Milwaukee, WI, USA) scanners with dedicated phased-array coil usually in combination with table-embedded spine coils. We performed abdominal and chest MRI following Institutional guidelines. Each examination was tailored to the patient clinical feature (body mass index) and the body region to be studied.

### Image interpretation

Two breast radiologists (A.T., M.C.), with more than 5 and 10 years of experience in breast MRI respectively, reviewed the MR images with detected breast findings and recorded the characteristic of the breast findings following the breast imaging reporting and data system (BI-RADS) MRI lexicon (American college of radiology breast imaging reporting and data system (BI-RADS) Atlas 2013). The two radiologists were blinded to clinical outcomes and previous breast imaging evaluation. For any patient who had more than one breast finding, each finding was included in the study. A single breast finding was considered for any patient with more than one simple breast cyst. For each body MRI with a breast finding, one radiologist (B.B.) reviewed the MRI report to evaluate any recommendations for additional work-up. The same radiologist assessed in the electronic medical record the availability of clinical follow-up and/or previous MRI examinations for comparison, or results of any additional imaging and histopathological work-up at our Institution for at least 6 months after the body MRI to evaluate the clinical relevance of breast finding and to follow-up the lesion classified as benign. Any breast findings initially assigned to BI-RADS category 3 but previously evaluated and described in the electronic medical record was downgraded and assigned to BI-RADS category 2, as previously performed in other similar setting (Niell et al. 2015).

### Cost analysis

The costs of further imaging evaluation and procedures were calculated on the basis of the reimbursement proposed at our Institution (€120 for standard mammography; €120 for breast ultrasonography; €400 for breast MRI; €200 for ultrasonography breast core-needle biopsy; €200 for ultrasound-guided preoperative lesion marking) and were intended as costs theoretically paid by patients. All further breast imaging work-up, follow-up and procedures performed after body MRI recommendation was included in the cost analysis.

### Statistical analysis

A descriptive analysis was performed among the patient population and corresponding MRI characteristic included in the study (patient age, percentage of breast tissue visible, MR magnetic field, type of MRI (abdominal or chest), the FOV size, the type of the contrast-media, number of examinations with diffusion-weighted images). We compared the presence of a breast finding and subsequent recommendations for further imaging and procedures between the groups of patients that performed abdominal and chest MRI, between the groups of patients that performed contrast-enhanced MRI and non-contrast enhanced MRI using the Fisher’s exact test. A p value of less than 0.05 was considered statistically significant significance (MedCalc Software bvba. Acacialaan 22, Ostend, Belgium) and we calculated 95 % exact confidence intervals (CIs). A clinically important finding in the breast was considered to be a cancer.

## Results

### Breast findings

440 body MRI examinations of 440 women (mean age: 57 ± 20 years) included breast tissue in the field-of-view (FOV range in abdominal MRI in millimetres: 340 × 276 to 400 × 337; FOV range in chest MRI: 350 × 295 to 380 × 344). Among the 440 body MRI examinations, 302 body MRI were performed on a 1.5 T scanner and 138 body MRI were performed on a 3.0 T scanner. In 201 patients (45.7 %), MRI showed more than 50 % of breast tissue, whereas in 239 patients (54.3 %) showed less than 50 % of breast tissue. MRI indications were: N = 216 liver imaging, N = 68 pancreatic imaging, N = 32 renal imaging, N = 17 adrenal imaging and N = 14 MR enterography; N = 66 mediastinum and N = 27 thoracic wall imaging. In 286 patients, body MRI was performed with intravenous contrast material injection; in 145 patients was performed gadoxetic acid enhanced MRI, in 109 patients was performed gadobenate dimeglumine-enhanced and in 32 patients gadoteridolo enhanced MRI.

Breast findings were identified in 41 of 440 patients (9.3 %). All these patients had a single breast finding. Table 1 shows body MRI with breast finding by BI-RADS category. Of the 41 patients with breast findings detected at body MRI, 25 patients (59.5 %) were categorized as BI-RADS 2; among these, three patients (12.0 %) were downgraded from an initial BI-RADS 3 or 4 by previous imaging work-up or review of the electronic medical record. Among the 25 findings classified as BI-RADS 2, the most common included simple breast cysts [88.0 % (22 of 25)]; previously known round or oval smoothly marginated hypervascular masses with some fat inside were the other benign findings [12.0 % (3 of 25)].

**Table 1:** Body MRI findings classified using BI-RADS category with corresponding MRI characteristics

BI-RADS category (0–4)	Number of patient with breast incidental findings	Breast incidental finding (%)	MRI characteristic
BI-RADS 2 + 3+4	41	100	
BI-RADS 2	25	59.5	
	22	88.0	Rounded lesion hyperintense on T2-weighted or STIR sequences
	3	12.0	Rounded/oval circumscribed mass previously described or evaluated
BI-RADS 3	13	30.9	
	8	61.5	Rounded/oval circumscribed mass not previously described or evaluated
	4	30.8	Oval mass
	1	7.7	T2-weighted or STIR hyperintense irregular lesion
BI-RADS 4	3	7.1	
	2	66.7	Suspicious enhancing mass
	1	33.3	Architectural distortion

The MRI characteristics represent the imaging characteristics of the incidental breast finding detected on body MRI examinations included in the study

A total of 16 breast incidental findings were categorized as BI-RADS 3 and 4, and the most common imaging findings included breast enhancing masses detected after contrast injection [87.5 % (14 of 16)]. The others imaging finding of BI-RADS 3 and 4 are described in Table 1.

**Fig. 2:**
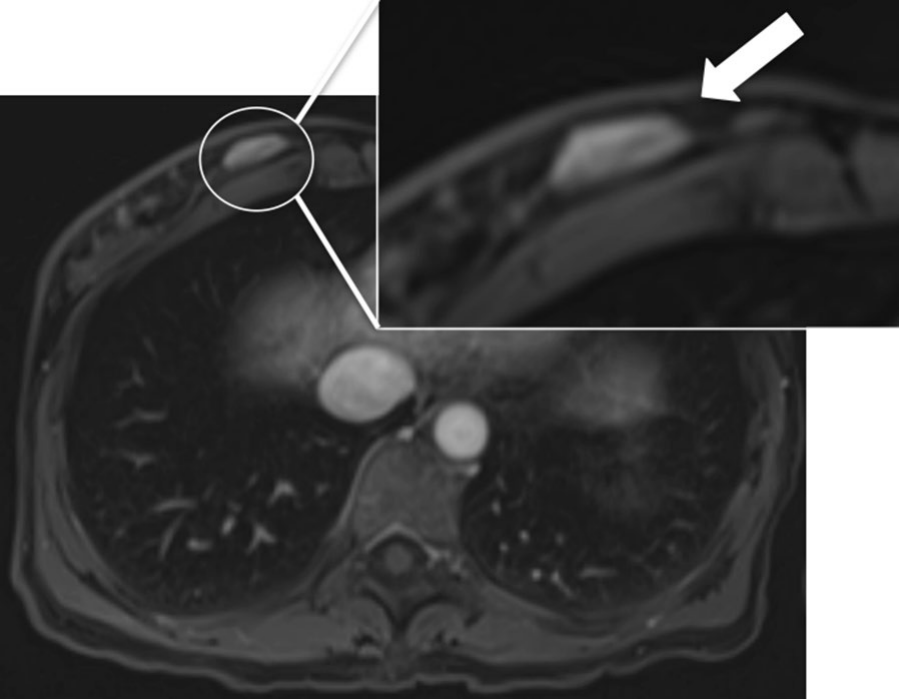
Examples of breast findings by BI-RADS category. A 49-year-woman underwent abdominal MRI for liver evaluation. Three-dimensional dynamic axial volumetric interpolated breath-hold examination image after intravenous power injection of 0.025 mmol/kg of gadoxetic acid (Primovist) at 60 s. Less than 50 % of the breast tissue was visible at abdominal MRI. An oval nodule (*white circle*) was found in the inner quadrant of the right breast (20 mm). The breast finding was first categorized as BI-RADS RM 3. After electronic medical record review this finding was downgraded as BI-RADS RM 2 (known fibrolipoma). In the *upper right corner* is highlighted the breast findings (*white arrow*)

**Fig. 3:**
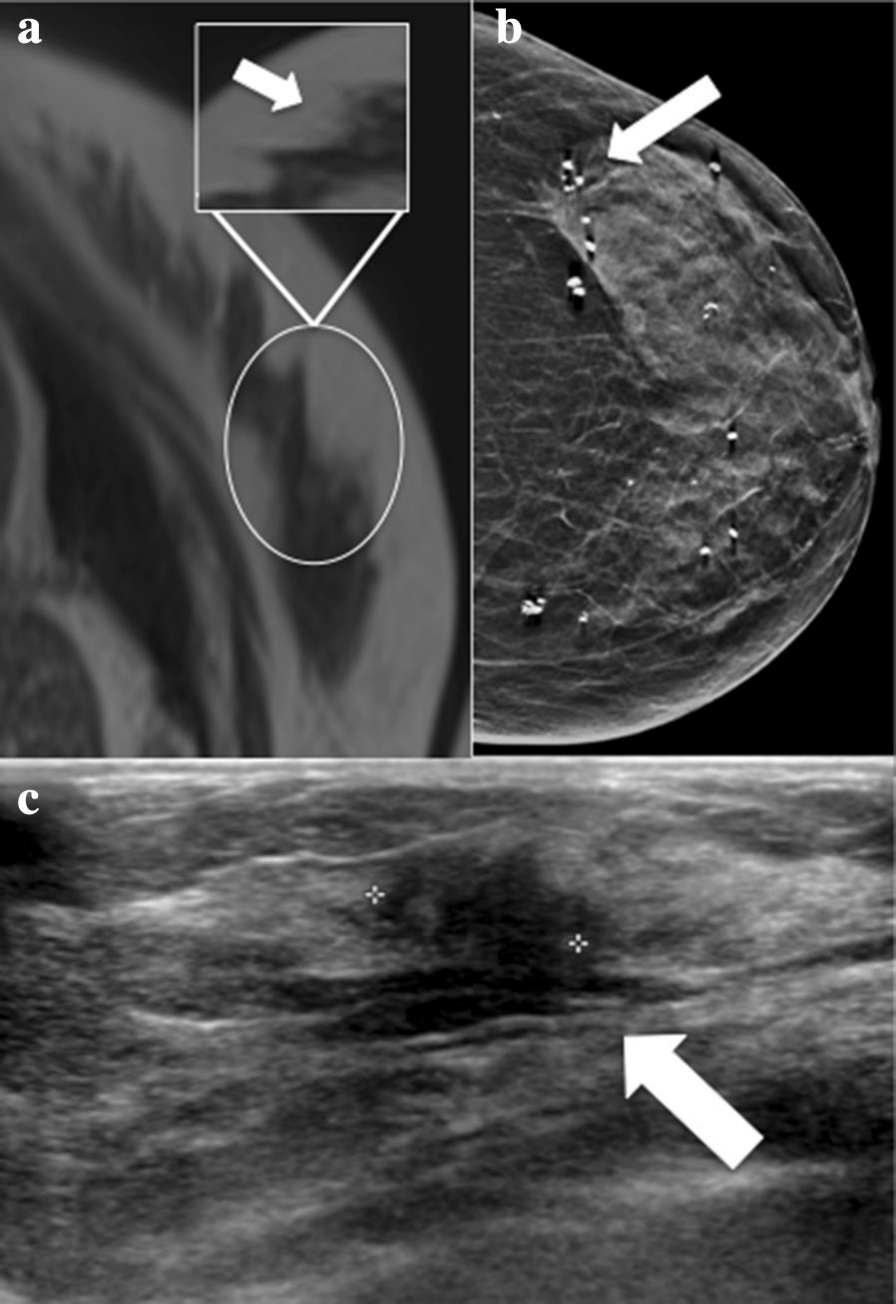
Examples of breast incidental findings by BI-RADS category. **a** A 75-year-woman (Patient 1 in Table 3) underwent MR cholangiopancreatography. More than 50 % of the breast tissue was visible at abdominal MRI. A suspicious architectural distortion (*white circle*) was found in the outer quadrants of the left breast (BI-RADS RM 4). In the *box* is highlighted the breast findings. **b** Left full-filed digital mammography shows an area of architectural distortion (*white arrow*) in the outer quadrants. **c** Corresponding US *image* shows a mass with indistinct borders in the left breast at 2 o’clock position (15 mm diameter). The US-guided core needle biopsy confirmed a B5b lesion (invasive ductal carcinoma)

**Fig. 4:**
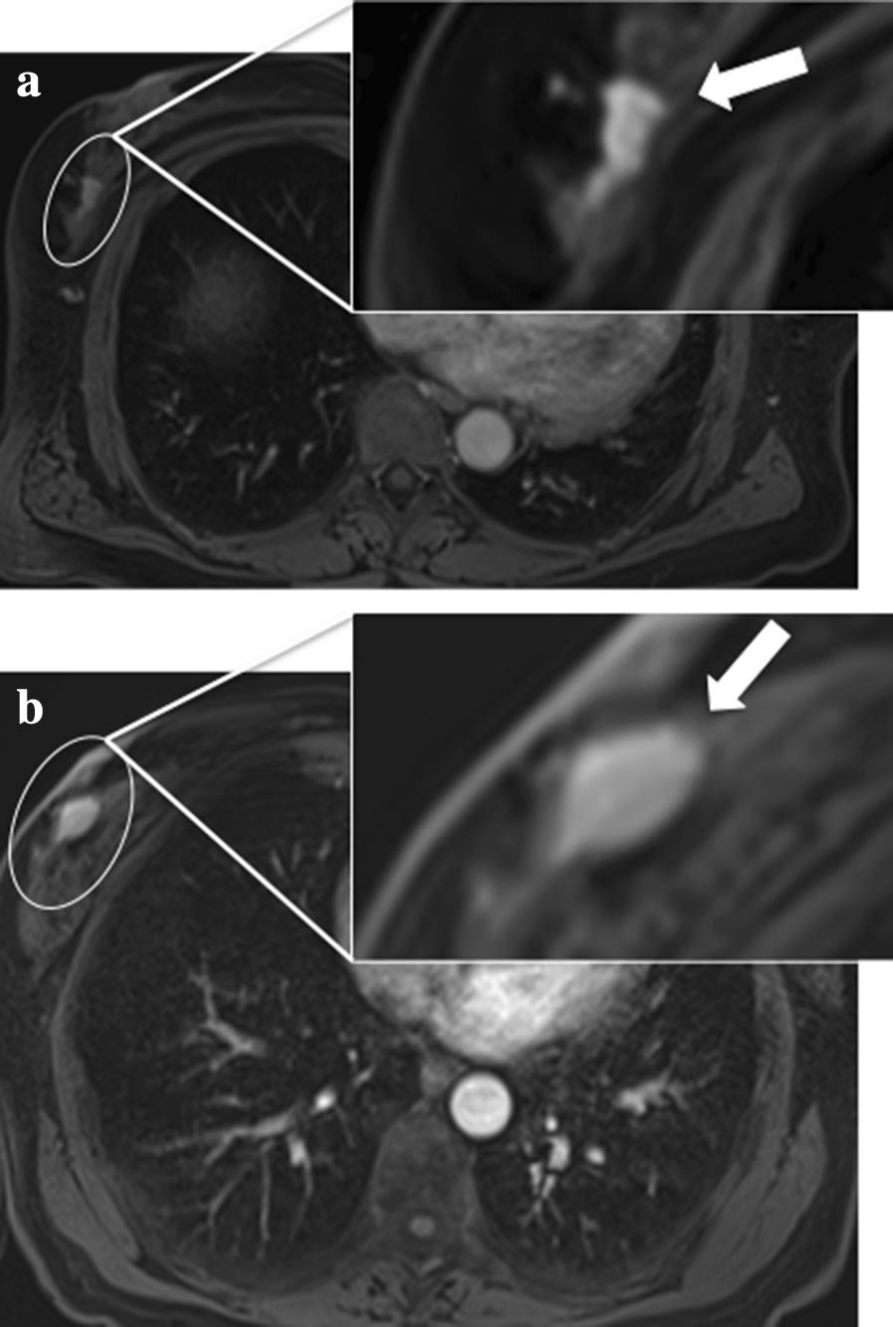
Examples of breast incidental findings by BI-RADS category. **a** A 67-year-woman (Patient 2 in Table 3) underwent abdominal MRI for follow-up of a cystic lesion of the head of the pancreas. Three-dimensional dynamic axial volumetric interpolated breath-hold examination image after intravenous power injection of 0.05 mmol/kg of Gadobenate dimeglumine (Multihance) at 60 s. More than 50 % of the breast tissue was visible at abdominal MRI. A suspicious mass (*white circle*) was found in the outer quadrants of the right breast (BI-RADS RM 4) The US-guided core needle biopsy confirmed a B5b lesion (Invasive Ductal Carcinoma). In the *upper right corner* is highlighted the breast findings (*white arrow*). **b** A 58-year-woman (Patient 3 in Table 3) underwent abdominal MRI for liver evaluation. Three-dimensional dynamic axial volumetric interpolated breath-hold examination image after intravenous power injection of 0.025 mmol/kg of gadoxetic acid (Primovist) at 60 s. More than 50 % of the breast tissue was visible at abdominal MRI. A suspicious mass (*white circle*) was found in the outer quadrants of the right breast (BI-RADS RM 4). This patient had a previously known breast cancer in the outer quadrants of the right breast, corresponding to the breast finding detected on MR images. In the *upper right corner* is highlighted the breast findings (*white arrow*)

Among the 41 patients with breast findings, 34 patients [82.9 % (34 of 41)] had breast finding identified during abdominal MRI and seven patients [17.1 % (7 of 41)] had breast finding identified during chest MRI (Table 2). Examples of patients with breast findings are shown in Figs. 2, 3 and 4.

**Table 2:** Breast incidental finding in abdominal and chest MRI examinations

Incidental finding by BI-RADS category	Chest MRI (N = 93)			Abdominal MRI (N = 347)		
	Contrast-enhanced MRI	Non-enhanced MRI	Total (%)	Contrast-enhanced MRI	Non-enhanced MRI	Total (%)
Total	5	2	7 (7.5 %)	19	15	34 (9.8 %)
BI-RADS 2	4	2	6 (6.4 %)	4	14	18 (5.2 %)
BI-RADS 3	1	Nf	1 (1.1 %)	12	Nf	12 (3.5 %)
BI-RADS 4	Nf	Nf	Nf	2	1	3 (0.9 %)

Body (chest and abdominal) MRI included in the study N = 440

*nf* not found

Of the 347 patients who underwent abdominal MRI, 34 patients (9.8 %) had a breast finding and of the 93 patients who underwent chest MRI, seven patients (7.5 %) had a breast finding (p = 0.69).

### Further imaging recommendation and clinical relevance

Of the 440 body MRI examinations, 16 patients (3.6 % [95 % CI 1.6 %, 5.6 %]) had a recommendation for an additional imaging to be performed for further evaluation of a breast finding. Of these 16 patients, 13 patients (81 %) were evaluated as BI-RADS category 3 and three patients (19 %) were evaluated as BI-RADS 4. Patient with BI-RADS 2 findings did no receive recommendation for additional imaging. Only one patient with chest MRI received additional imaging recommendation for a BI-RADS 3 finding.

Of the 347 patients who underwent abdominal MRI, 15 patients (4.3 %) received additional recommendation for further imaging and procedures. Of the 93 patients who underwent chest MRI, one patient (1.1 %) received additional recommendation for further imaging for a BI-RADS 3 breast finding (p = 0.212).

Patients with contrast-enhanced MRI were significantly more likely to have a recommendation for additional imaging evaluation compared with patients undergoing non contrast-enhanced MRI (non-enhanced MRI, one patients; contrast-enhanced MRI, 15 patients; p = 0.015).

All patients who underwent contrast-enhanced MRI were, on average, aged 63.5 years (age range, 19–84 years) compared with 59.9 years (age range, 26–88 years) for all patients with non contrast-enhanced MRI (p = 0.0063).

All patients who underwent abdominal MRI were, on average, aged 61.8 years (age range, 19–86 years) compared with 51.2 years (age range, 23–88 years) for all patients with chest MRI (p < 0.0001).

Overall, 3.6 % (16 of 440) of female patients with breast tissue on body MRI received a recommended additional imaging examinations for further evaluation of a probably benign (BI-RADS 3*)* or suspicious (BI-RADS 4) breast finding by using body MRI. Of the 14 patients [34.1 % (14 of 41)] with breast finding who underwent the recommended additional imaging on the basis of the body MRI findings, 11 patients (78.6 %) were BI-RADS 3 and three patients (21.4 %) were BI-RADS 4. Two patients [12.5 % (2 of 16)] with BI-RADS 3 were lost at follow-up and did not undergo the additional imaging recommended. No additional imaging was recommended for incidental breast findings categorized as BI-RADS 2.

Clinical relevance of breast incidental finding was available for 14 patients (follow-up interval range, 6–14 months). After undergoing additional imaging examinations, three patients [21.4 % (3 of 14)] had a clinically important finding. Two patients had a previously unknown invasive breast cancer (invasive ductal carcinoma) and one patient had a previously known invasive breast cancer (invasive ductal carcinoma) (Table 3). Therefore, 18.7 % (3 of 16) of women undergoing further imaging work-up because of recommendation of breast incidental finding with body MRI had a clinically important finding in the breast.

**Table 3:** Patients with clinical relevant breast finding

Patient	Age (years)	Previous history of cancer	MRI performed	Field of view	Contrast media	Clinical indication	BI-RADS category of the incidental finding at body MRI	Final diagnosis at surgery
1	75	Previous history of breast cancer	Abdominal MRI	380 × 261	Not used	MR cholangiography	BI-RADS 4	Invasive ductal carcinoma
2	67	No history	Abdominal MRI	380 × 380	Gadobenate dimeglumine	Pancreatic MRI	BI-RADS 4	Invasive ductal carcinoma
3	58	No History	Abdominal MRI	308 × 380	Gadoxetic acid	Liver MRI	BI-RADS 4	Invasive ductal carcinoma

Three of 440 patients (0.7 %) had a clinically important finding identified with body MRI, and these included two patients who had previously unknown invasive breast cancer.

One patient with a clinically important finding had an indication for liver MRI. One patient had an indication for pancreatic imaging and the other patient had an indication for MR cholangiography. One of the three patients with a malignant breast lesion had a previous history of breast cancer. The remaining two patients had no previous history of malignancies (Table 3).

### Cost of further imaging and procedures

Of the 16 patients with recommendations for additional imaging, four patients had one additional imaging study recommended, and four patients had two additional imaging studies recommended, one patient had three additional imaging recommendation, five patients had one additional imaging and one procedures recommendation, and two patient had two additional imaging and two procedures recommendations, which totalled 33 additional imaging studies and procedures that were recommended (Table 4). Enhancing mass accounted for 94 % (31 of 33) of these recommendations, including 19 of the 21 recommendations for breast ultrasonographic imaging. Of the imaging studies that were performed, ultrasound examinations were the majority [61 % (19 of 31)] of additional imaging examinations recommended for diagnosis of newly identified breast findings.

**Table 4:** Additional imaging studies and work-up recommended and performed because of breast findings at body MRI and associated additional costs

	FFDM	US	US core-needle biopsy	Ultrasound-guided preoperative lesion marking	Number of additional imaging studies and work-up recommended	Additional costs for imaging studies and work-up performed (Euros)
Chest MRI		2			2	240
Abdominal MRI	3	19◆	7	2	31◆	4200
Total	3	21◆	7	2	33◆	4440

*FFDM* full-field digital mammography, *US* ultrasound

◆Two ultrasound examinations were not performed (patients lost at follow-up)

Subsequent imaging studies and additional costs resulted are shown in Table 4.

The total costs for all subsequent imaging studies and procedures performed as a result of a body MRI recommendation for additional imaging evaluation and procedures was €4440. The entire procedures constituted the 9 % (€400 of €4440) of the additional costs (Table 4). The 2015 costs at our Institution of contrast-enhanced abdominal or chest MRI examination is €450, non contrast-enhanced is €350. Therefore, for each of the 440 body MRI examinations included in this study an average additional cost of €10.1 was attributable to the additional imaging recommended.

Sub-analyses of cost were performed for the 16 of 41 body MRI examinations with breast findings that resulted in recommendations for additional imaging evaluation. The 2015 costs at our Institution of contrast-enhanced abdominal or chest MRI examination is €450, non contrast-enhanced is €350. Therefore, for each of the 41 body MRI with incidental breast finding performed in this study, an average additional cost of €108.3 was attributable to the additional imaging recommended.

## Discussion

Breast cancer is the most frequent cancer in women and is the leading cause of cancer-related deaths of European women (Senkus et al. 2015; Sardanelli et al. 2010; Mann et al. 2015). Breast MRI is a highly sensitive imaging method for breast cancer detection (Senkus et al. 2015; Sardanelli et al. 2010; Mann et al. 2015; Morrow et al. 2011). For breast cancer screening, abbreviated and non-standard MRI protocols have been evaluated (Kuhl et al. 2014; Mango et al. 2015; Carbonaro et al. 2012).

In clinical practice, a significant amount of breast tissue is commonly visible during body MRI. Therefore, given the high sensitivity of MRI, there could be some breast findings visible in the field-of-view. In this study, we aimed to assess breast findings in women undergoing body MRI. To the best of our knowledge, this is the first study assessing the presence of breast lesions incidentally detected on abdominal and chest MRI. We found that, among female patients undergoing body MRI, at least 3.6 % had a recommendation for additional imaging examinations and procedures due to an incidental breast finding, and 0.7 % had a clinically relevant incidental breast finding. Notably, we found three invasive breast cancers (invasive ductal carcinoma). One patient undergoing non contrast-enhanced abdominal MRI had an incidental breast finding that received recommendation for further imaging work-up and this patient was revealed to have a breast cancer. The relative high prevalence of breast cancer in the group of patients undergoing non contrast-enhanced body MRI compared to the group of patients undergoing contrast-enhanced MRI could be related to the high intrinsic sensitivity of contrast-enhanced body MRI that leads to a higher detection of breast findings compared to the other group. For the same reason, the majority of breast findings detected during contrast-enhanced MRI had higher BI-RADS category compared to the breast findings detected during non-contrast-enhanced MRI. However, this consideration has to be confirmed on larger series. The European Society of Breast Imaging (EUSOBI) guidelines acknowledge that breast MRI should be performed routinely with contrast agent for diagnostic value (Mann et al. 2015), indeed, we do not believe that non contrast-enhanced body MRI could be an accurate imaging modality for breast cancer detection. In addition, our results do not support the use of body MRI as an additional screening modality for breast cancer detection, but highlight the possibility to find a breast lesion on body MRI.

The prevalence of our findings is consistent with other cross-sectional imaging examinations: the detection of clinically relevant incidental breast findings varies from 0.3 % on computed tomography of the body (Monzawa et al. 2013), to 6 % on PET-CT (Benveniste et al. 2015). Regarding previous studies dealing with incidental breast finding on MRI, it has been evaluated the prevalence of breast cysts in whole-body MRI, which was 3.5 % for patients over the age of 50 years (Cieszanowski et al. 2014). However, breast cysts are not clinically relevant. A recent report (Bamberg et al. 2015) shows the design of the MRI Study of the German National Cohort with a particular mention about incidental findings: a major challenge is that the whole-body unenhanced MR protocol is different from that used on diagnostic application, and, for examples, breast cancer cannot be detected with reasonable specificity. Differently from previous studies, we reviewed both non-contrast and contrast-enhanced abdominal and chest MRI examinations and we were able to detect not only breast cysts but more suspicious breast incidental findings too.

Regarding the percentage of breast tissue visible in our study population during MRI, the majority of patients had less than 50 % of breast tissue visible. Therefore, it is possible that other breast findings would have been visible using a larger FOV. Although this study is formally retrospective, the MR images review allowed a simile-prospective reading of all images with a blind assessment of the breast tissue that would have not been feasible with the review of the MR report alone. This technique of reading was very time consuming, but we believe that increase and strengthen the results of the study. Regarding costs, we found that additional imaging and procedures for breast findings contributed an increased cost of €108.3 for each patient with incidental findings. However, additional recommendations for breast finding do not increase cost significantly per women undergoing body MRI with breast tissue visible; indeed, an average additional cost of €10.61 was totalled for each patient included in the study. We acknowledge that the costs of additional recommendation lacks of the costs of the surgical procedures performed for breast cancer therapy, underestimating the average additional costs per patient. On the contrary, we could not perform a cost-analysis about the saving costs for early breast cancer detection.

Our study has other limitations. First, we did not assess the correlation between detection of breast finding and clinical characteristic and technical parameters. In addition, we did not evaluate the histological size of the breast cancers because the evaluation of the incidence of breast cancers during body MRI and its correlation with breast cancer characteristics was not the purpose of our study. Finally, the study design could have underestimated the number of total breast findings due to inclusion of patients with percentage of breast tissue visible and not the whole breast.

## Conclusions

In conclusion, at least 3.6 % of women undergoing body MRI had a breast incidental finding, and 18.7 % of these patients had a clinically relevant incidental breast finding. Further imaging work-up and procedures recommended did not increase significantly the cost per women undergoing body MRI.

## Abbreviations

MRI: magnetic resonance imaging
CT: computed tomography
FOV: field-of-view
FDG PET/CT: (18) F-fluorodeoxyglucose positron emission tomography–computed tomography
CIs: confidence intervals
BI-RADS: breast imaging reporting and data system
EUSOBI: European Society of Breast Imaging
